# Identifying mislabeled and contaminated DNA methylation microarray data: an extended quality control toolset with examples from GEO

**DOI:** 10.1186/s13148-018-0504-1

**Published:** 2018-06-01

**Authors:** Jonathan A. Heiss, Allan C. Just

**Affiliations:** Department of Environmental Medicine and Public Health, Icahn School of Medicine at Mount Sinai, One Gustave L. Levy Place, Box 1057, New York, 10029 NY USA

**Keywords:** DNA methylation, Epigenomics, Infinium, 450K, EPIC, Quality control, Contamination, Mislabeling, Data cleaning

## Abstract

**Background:**

Mislabeled, contaminated or poorly performing samples can threaten power in methylation microarray analyses or even result in spurious associations. We describe a set of quality checks for the popular Illumina 450K and EPIC microarrays to identify problematic samples and demonstrate their application in publicly available datasets.

**Methods:**

Quality checks implemented here include 17 control metrics defined by the manufacturer, a sex check to detect mislabeled sex-discordant samples, and both an identity check for fingerprinting sample donors and a measure of sample contamination based on probes querying high-frequency SNPs. These checks were tested on 80 datasets comprising 8327 samples run on the 450K microarray from the GEO repository.

**Results:**

Nine hundred forty samples were flagged by at least one control metric and 133 samples from 20 datasets were assigned the wrong sex. In a dataset in which a subset of samples appear contaminated with a single source of DNA, we demonstrate that our measure based on outliers among SNP probes was strongly correlated (> 0.95) with another independent measure of contamination.

**Conclusions:**

A more complete examination of samples that may be mislabeled, contaminated, or have poor performance due to technical problems will improve downstream analyses and replication of findings. We demonstrate that quality control problems are prevalent in a public repository of DNA methylation data. We advocate for a more thorough quality control workflow in epigenome-wide association studies and provide a software package to perform the checks described in this work. Reproducible code and supplementary material are available at 10.5281/zenodo.1172730.

## Background

The number of epigenome-wide association studies in epigenetic epidemiology is growing rapidly, facilitated by popular microarray platforms like the Infinium 450K and EPIC chips, which offer broad coverage and precise quantification of DNA methylation. Whereas the literature about preprocessing and statistical analysis of microarray data is extensive [1, 2], the need for upstream quality control (QC) to ensure robust and reproducible results has received much less attention. Epigenome-wide association studies usually start with a quality control step, often involving discarding individual probes or entire samples with too many high detection *p* values (resulting from a low signal-to-noise ratio of fluorescence intensities), probes with too few beads, or discarding subsets of probes that are considered unreliable based on design features (e.g., those being cross-reactive or close to SNPs). However, there is a lot of heterogeneity in which checks are undertaken and how criteria are applied, as seen in the methods reported across a large consortium of birth cohort studies [3]. Furthermore, quality checks that go beyond to catch other types of problematic samples are not yet standard procedure. There are many reasons microarray experiments might fail: starting with low-quality DNA input, an incomplete bisulfite conversion, or a failure of the other experimental steps in the Infinium assay. These issues can result in distorted methylation patterns, which may be sometimes more or less apparent. Another common source of errors is sample mislabeling, especially in large multi-center studies where the chain of custody of the sample collection process is long and mistakes can happen at every step. Mislabeling creates mismatches between epi- and phenotype, thereby obfuscating genuine associations or even producing spurious ones. The prevalence of mislabeling was recently demonstrated in 70 publicly available gene expression datasets hosted in the Gene Expression Omnibus (GEO) repository: Toker et al. used genes known to be differentially expressed between sexes to infer the sex of the sample donors and to compare it to the recorded metadata. They found that 32 of the datasets contained discrepancies in a subset of the samples [4]. Finally, sample contamination with foreign DNA may arise accidentally in the laboratory or from complex sampling procedures (e.g., contamination of cord blood or placental tissue with maternal blood [5]) and analytic methods are needed to identify and quantify contamination.

Having found mislabeled and contaminated samples in our own DNA methylation datasets, we developed a software package for the R programming language named ewastools aiming to facilitate quality control and statistical analysis of datasets generated from the Illumina Infinium BeadChip platforms (both 450K and the newer EPIC). In order to test the package functionality, we decided to apply it to a range of datasets from the public Gene Expression Omnibus (GEO) repository. The results show that low-quality samples, mislabeling, and contamination of samples are widespread issues. It is our hope that the ewastools package will help researchers to extend and improve the quality control workflow in epigenome-wide association studies.

## Methods

### Datasets

The search for DNA methylation datasets was limited to the popular Gene Expression Omnibus repository. We selected datasets meeting the following criteria: they had to be submitted before January 2018; samples had to be run on the Illumina Infinium HumanMethylation450 BeadChip (GEO Accession GPL13534); the sex of the sample donors had to be provided in the metadata; and raw data had to be provided in the form of.idat files. Datasets containing only preprocessed data were not included as they may no longer contain QC probes and the lack of a standard format makes their analysis difficult to automate. While our ewastools package offers the same functionality for the EPIC chip, our selection was limited to the predecessor 450K chip as the two platforms differ in the number of SNP probes which are essential for the checks described below. Datasets meeting these criteria were identified using the Entrez Programming Utilities (http://eutils.ncbi.nlm.nih.gov/). From this list we manually excluded datasets based on the description and metadata provided in GEO: we dropped datasets involving samples from tumor tissue or cultivated cell lines because tumor cells can show extensive epigenetic mutations related to defects in the DNA methylation maintenance apparatus [6], whereas for the latter it is unclear to what degree methylation profiles reflect the natural/in vivo state of the cell types rather than an epigenome of manipulation [7]; we dropped placenta samples because they could either be of maternal or fetal origin, as well as sperm samples as the cells are haploid; we dropped FFPE (formalin-fixed, paraffin-embedded) samples as their preparation follows a different procedure than for fresh tissue samples and the DNA is often of lower quality; lastly, we excluded datasets measuring 5-hydroxymethyl-cytosine. Eventually, a total of 80 datasets comprising 8327 samples remained, representing a broad range of tissues and cell lines (peripheral blood, cord blood, saliva, liver, muscle, cartilage, etc.).

### Quantification of methylation levels

Treating DNA with sodium bisulfite converts cytosine to thymine except for 5-methylcytosine, which is protected by the added methyl-group. In combination with subsequent whole-genome amplification, the proportion of unmethylated to methylated DNA strands in the DNA input is translated into differences in abundance of distinct PCR (polymerase chain reaction) products, which, for the sake of simplicity, are here still referred to as (un)methylated. The 450K chip employs 50 base pairs long probes complimentary to the targeted loci. Unmethylated and methylated strands are targeted by separate probes or color channels. Their abundance is quantified by hybridization with the corresponding probes, a subsequent single-base extension step with dye-linked nucleotides and measurement of the resulting fluorescence intensity. Thus, two data points are available for each CpG site *i*, the intensity for unmethylated and methylated strands, *U*_*i*_ and *M*_*i*_, respectively. Here, intensities were corrected for dye bias using RELIC [8], but not normalized. The proportion of methylated strands was then estimated as $\frac {M_{i}}{M_{i}+U_{i}}$, commonly referred to as the *β*-value.

### Quality checks

Four kinds of quality checks are implemented in the ewastools package: an evaluation of control metrics monitoring the various experimental steps such as bisulfite conversion or staining; a sex check comparing the actual sex of the sample donors to the records; an identity check for fingerprinting sample donors; and detection of contaminated samples using outliers among the 65 probes querying high-frequency SNPs.

The first check, implemented by the function control_metrics, evaluates 17 control metrics calculated from dedicated control probes placed on each assay. A description of these metrics, together with default thresholds to flag problematic samples, is provided in the BeadArray Controls Reporter Software Guide available from the Illumina support website. Similar functions to evaluate the control probes visually are provided in the minfi [9], RnBeads [10] and shinyMethyl [11] R packages. Not all flagged samples necessarily failed nor do these metrics indicate potential upstream issues, e.g. whether the DNA quality was low to begin with. All 8327 samples were screened in this first check.

There are 65 probes placed on the 450K chip querying high-frequency SNPs (with 59 of these on the EPIC chip; their probe identifiers start with “rs”). Just as for CpG sites, a *β*-value is calculated for each SNP locus, based on fluorescence intensities from two probes targeting either the wild type or the common mutant variant. These *β*-values usually fall into one of three disjunct clusters, corresponding to the heterozygous and the two homozygous genotypes (AB, AA, or BB). The specific combination of SNPs across these 65 probes serves as a genetic fingerprint: fingerprints of samples from the same donor match but differ between individuals – with the exception of monozygotic twins – thereby enabling one to check for discrepancies with the metadata. Genotype calling is handled by call_genotypes. This function pools *β*-values of all 65 SNP probes across samples to train a mixture model with four components: three Beta distributions, each representing one genotype, and one uniform distribution representing outliers. Subsequently, posterior probabilities are computed and forwarded to check_snp_agreement. Using the posterior probabilities as soft classification, pairwise agreement of fingerprints is assessed by counting the number of SNP probes for which two samples possess the same genotype, divided by the total number of SNP probes after those classified as outliers were excluded (consistent with the soft genotype calling, a SNP might be partially classified as outlier and therefore be only partially excluded). Mislabeling constitutes either as unexpected disagreement between samples that are supposed to come from the same individual, or unexpected agreement between samples supposed to come from different individuals. A list of such instances, here termed conflicts, is returned by check_snp_agreement. This second quality check was applied to a dataset comprised of 150 pairs of monozygotic twins (in total 300 samples and 10 technical replicates resulting in 47,895 pairwise comparisons).

The total intensity *T*_*i*_=*U*_*i*_+*M*_*i*_ has been shown to be sensitive to copy number aberrations [12]. By exploiting the natural difference in allosomal copy number, this can be used to detect sex-mismatches. There are 11,232 and 413 probes on the 450K chip targeting the X and Y chromosome, respectively. The function check_sex computes for each sample *n* the average total intensities of all probes targeting either chromosome, $\bar {T}^{X}_{n}$ and $\bar {T}^{Y}_{n}\!\!$, respectively. In order to account for differences in post-amplification DNA concentration, $\bar {T}^{X}_{n}$ and $\bar {T}^{Y}_{n}$ are normalized by the average total intensity across all autosomal probes which leads to more compact clusters in visualizations. Thresholds to discriminate between both sexes are determined by the Hodges-Lehmann estimator (median of all pairwise male/female averages) for $\bar {T}^{X}_{n}$ and $\bar {T}^{Y}_{n}$ separately. This robust approach was chosen over other sex determination functions provided in the minfi [9], RnBeads [10] and shinyMethyl [11] packages exactly because of the potential of sex-mismatches and allosomal outliers being present in the dataset. All 8327 samples were screened in this third check.

When exploring the data, one dataset in particular, here referred to as dataset **E**, caught our attention. Plotting $\bar {T}^{X}_{n}$ against $\bar {T}^{Y}_{n}\!\!$, most samples—aside from a few mislabeled ones—clustered as expected, but a subset of samples from female donors were protruding from the cluster center in the direction of the male cluster center (Fig. 3b): such a pattern is indicative of sample contamination and more specifically, it is compatible with the hypothesis of the foreign DNA coming from a male source. This does not imply that only female samples were affected: in the case of a male/male DNA mix, both allosomes would still show a methylation profile typical for males, only in the case of a female/male mix would both allosomes show methylation profiles atypical for either males or females. With increasing degree of contamination, such samples would be further away from the female cluster center and closer to the male cluster center.

In order to confirm this hypothesis, we turned again to the 65 SNP probes. Normally, their *β*-values fall, according to the underlying genotype, into one of three disjunct clusters. In dataset **E** however, many *β*-values fell in-between these three clusters (plotting their histogram would show three peaks no longer completely disjunct), a pattern one would expect to see when mixing two genotypes (the same way *β*-values of heterozygous AB individuals scatter around 0.5, as they possess a 50:50 mixture of A and B alleles). We translated our hypothesis in a generative statistical model with the following likelihood function $\prod _{n=1}^{N}\prod _{j=1}^{65} \beta _{nj}\sim \mathcal {N}\left ((1-\gamma)\cdot \mu _{k_{nj}}+\gamma _{n}\cdot c_{j},(1-\gamma _{n})\cdot \sigma _{k_{nj}}\right)$ and parameters *n*∈ [ 1,*N*] (sample index), *j*∈ [ 1,65] (SNP probe index), *k*∈{AA,AB,BB} (the three possible genotypes), *k*_*nj*_ (genotype of sample *n* for probe *j*), *β*_*nj*_ (methylation level of sample *n* for SNP probe *j*), *σ*_*k*_ (standard deviation for *β*-values from genotype *k*), *c*_*j*_ (methylation level of SNP probe *j* in foreign DNA), and *γ*_*n*_ (degree of contamination of sample *n*). The parameters were estimated using the Metropolis algorithm implemented in the Julia programming language (code is provided the supplement). Comparing *γ*_*n*_ (degree of contamination) to $\bar {T}^{Y}_{n}$ for female samples allowed us to test if both agreed on ranking samples from least to most contaminated. This check was applied only to dataset **E**.

Our precise quantification of sample contamination in dataset **E** required the assumption that the foreign DNA came from a single source, which may be an unusual scenario. A simpler and more general test of sample contamination that can be applied to any dataset is implemented in the function snp_outliers. This function, again using the output of call_genotypes, computes *O*_*n*_, the average log odds from the 65 posterior probabilities from the outlier component from the mixture model described above. Thus, *O*_*n*_ captures how irregular the SNP *β*-values of sample *n* are, i.e., how much they deviate from the ideal trimodal distribution. *O*_*n*_ was compared to *γ*_*n*_ in dataset **E** in order to confirm that it did indeed capture sample contamination. Subsequently, all 8327 samples were screened in this fourth check.

## Results

**Fig. 1:**
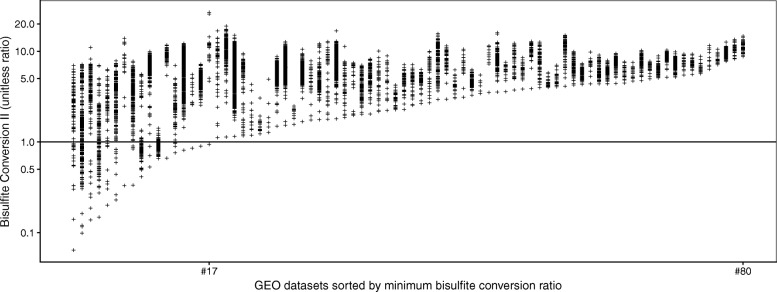
Distribution of a control metric monitoring bisulfite conversion. Samples below the manufacturer’s suggested threshold of 1 might be incompletely converted, leading to inaccurate estimates of methylation levels

Figure 1 shows the distribution of the “Bisulfite Conversion II” control metric, used to monitor successful bisulfite conversion, across the 80 datasets. Three hundred seventy-seven samples from 17 datasets fell below the default threshold specified by Illumina. Overall, 940 samples (11%) from 41 datasets (51%) were flagged by at least one of the 17 metrics in control_metrics, leaving 7387 samples that passed this first check. A summary is provided in Table 1. Many of these problematic samples would be overlooked when filtering out only samples with too many undetected probes or low overall fluorescence intensity, two popular criteria. Out of the 940 flagged samples, 432 had > 1*%* of probes with a detection *p* value above 0.01 and 217 were flagged by the getQC function from the minfi package, and 541 samples when considering both criteria.

**Table 1:** Summary of the number of samples flagged by each of the 17 control metrics outlined in the BeadArray controls reporter software guide. n.a.—not available

Metric	Passed	Flagged	n.a.
Restoration	8326	0	1
Staining green	7885	10	432
Staining red	7764	14	549
Extension green	8324	3	0
Extension red	8326	1	0
Hybridization high/medium	8326	1	0
Hybridization medium/low	8327	0	0
Target removal 1	8327	0	0
Target removal 2	8327	0	0
Bisulfite conversion I green	8317	10	0
Bisulfite conversion I red	8208	119	0
Bisulfite conversion II	7950	377	0
Specificity I green	8323	4	0
Specificity I red	8279	48	0
Specificity II	8326	1	0
Non-polymorphic green	7677	558	92
Non-polymorphic red	7917	318	92
Any of the above	7387	940	–

**Fig. 2:**
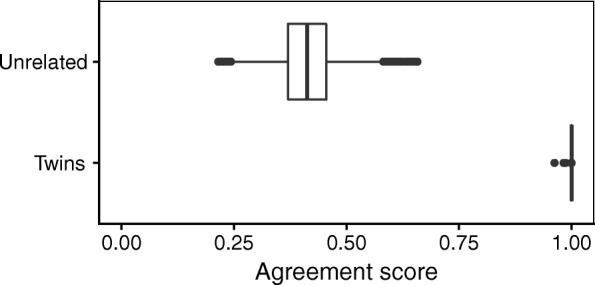
Pairwise agreement scores of genetic fingerprints in a dataset of monozygotic twins. There is a perfect segregation between twin and non-twin pairs

Figure 2 shows the result of the identity check in the dataset comprising 150 monozygotic twins. Pairwise agreement of SNP fingerprints between samples ranged from 0.96 to 1.00 for the 187 twin pairs (number is larger than 150 because of the technical replicates) and from 0.22 to 0.66 for the 47,708 non-twin pairs, thereby perfectly segregating both groups. For comparison, genotype calling by k-means clustering, as implemented in the wateRmelon package [13], produced non-identical fingerprints in 11 out of the 187 twin pairs, with one twin pair showing as many as 9 discordant SNPs.

**Fig. 3:**
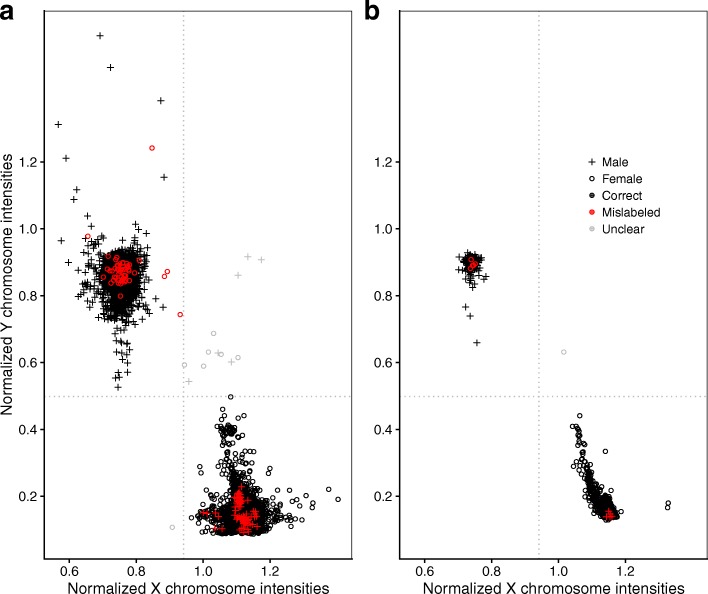
Average fluorescence intensities of probes targeting the X chromosome (*x*-axis) or targeting the Y chromosome (*y*-axis), each normalized versus the average fluorescence of autosomal probe intensities per sample. Dotted lines represent the Hodges-Lehmann estimators separating the male and female cluster centers. Samples that are discordant for sex relative to their metadata annotation are considered mislabeled and shown in red. Samples falling in the lower left or upper right quadrant are considered “unclear”. **a** All 8327 samples from all 80 datasets. **b** A single dataset (dataset **E**) with a spread in the female cluster indicating varying degrees of contamination with male DNA

The function check_sex estimated that 133 of the 8327 samples had been assigned the wrong sex yielding an error rate of 1.6%, with 13 cases being unclear, as the inferred sex was discordant between $\bar {T}^{X}_{n}$ and $\bar {T}^{Y}_{n}$ (Fig. 3a). Of the 80 datasets, 20 (25%) contained these sex-mismatched samples. These were unevenly distributed: the three highest error rates per study were 55% (#mistakes 38/#total sample size 69), 45% (5/11), and 34% (15/44). Excluding all samples that failed the control_metrics check and might have technical errors in measurement did not change sex-mismatched error rates substantially: out of the 7387 samples, 122 (1.7%) had been assigned the wrong sex with 10 unclear cases. Comparing with the predictions provided from minfi, there were, apart from the 13 unclear cases mentioned above, 66 female (according to metadata) samples from six datasets that were correctly classified by ewastools but misclassified by minfi.

**Fig. 4:**
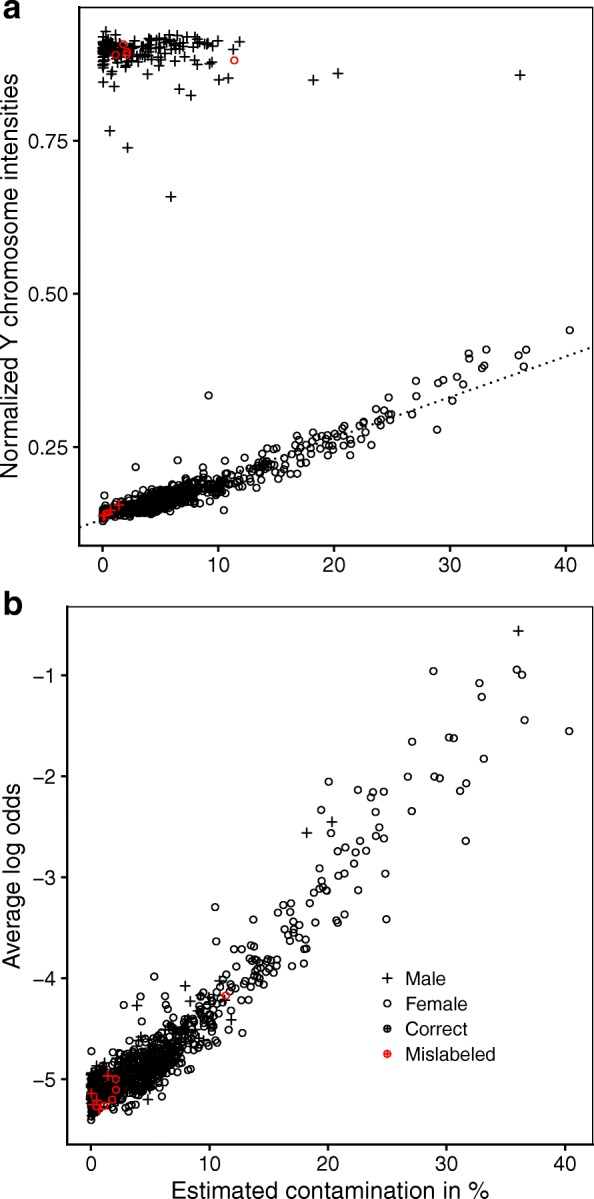
Evidence of contamination in dataset **E**. **a** *γ*_*n*_, the estimated degree of contamination, and $\bar {T}^{Y}_{n}$ are strongly correlated among females with a Pearson’s correlation coefficient *r*=0.953. **b** *γ*_*n*_ and *O*_*n*_, the average log odds of SNP probes being outliers, are strongly correlated as well (*r*=0.954), including both males and females

Estimating the degree of contamination *γ*_*n*_ for each sample in dataset **E**, we found that *γ*_*n*_ and $\bar {T}^{Y}_{n}$ were strongly correlated among females (Pearson’s correlation coefficient 0.953, 95% bootstrapped CI 0.934,0.965; Fig. 4a), confirming that both metrics agreed on ranking samples from least to most contaminated. Correlation was weaker for $\bar {T}^{X}_{n}$ (− 0.828, 95% CI − 0.898, − 0.748), suggesting that $\bar {T}^{Y}_{n}$ was the more sensitive metric of contamination in this situation. Males were not included in the calculation of these correlation coefficients because contamination would not affect $\bar {T}^{Y}_{n}$ nor $\bar {T}^{X}_{n}$ if our hypothesis of contamination coming from a single male source were correct. Even though *γ*_*n*_ was non-zero for most samples, we assume that only a subset of the samples were indeed contaminated, as those with the largest *γ*_*n*_ tended to be allocated next to each other on the 450K chips (not shown). *γ*_*n*_ and *O*_*n*_ for the same set of samples were strongly correlated as well (Pearson’s correlation coefficient 0.958, 95% bootstrapped CI 0.948,0.966), even when including males (0.954, 95% CI 0.944,0.961; Fig. 4b), suggesting that in this dataset *O*_*n*_, the average log odds of SNP probes being outliers, was a proxy for sample contamination not contingent on the sex of the sample donor or source of contamination (with the exception of contaminating DNA coming from another tissue of the same donor).

**Fig. 5:**
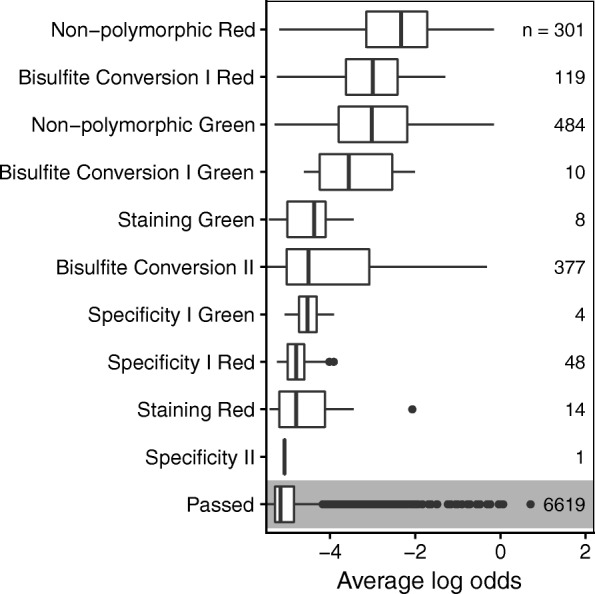
Boxplots of *O*_*n*_ (average log odds of being an outlier across the 65 SNP probes) as a measure of low technical performance for samples flagged by any of the 17 Illumina control metrics and samples passing all of them. Metrics are ordered by median *O*_*n*_ with the number of samples in each non-exclusive category indicated on the right. Flagged samples have in general higher values of *O*_*n*_ than samples that passed all checks, indicating that flagged samples are more likely to have poor performance characteristics

While the importance of a metric monitoring critical laboratory steps such as bisulfite conversion is obvious, the relevance of Illumina provided default thresholds for other metrics is less clear. Figure 5 shows the distribution of *O*_*n*_ among all samples that were flagged by the control_metrics checks versus the remainder that passed. We use *O*_*n*_ here as a measure of poor technical performance, rather than a measure of sample contamination, as the former would also contribute to a deviation of the SNP probes from the ideal trimodal distribution. Flagged samples had in general higher values of *O*_*n*_, indicating that such samples are indeed of concern.

## Discussion

The topic of 450K data quality has been addressed before. Among the issues being discussed are cross-reactive probes and probes possibly affected by nearby SNPs [14, 15], high detection *p* values and batch effects [16]. While these publications focus on probes with low fluorescence intensities or that are in general unreliable, or issues that affect ensembles of samples, our work is in contrast mainly concerned with the identification of individual problematic samples resulting from failed experiments, mislabeling or contamination. Due to the often small effect sizes, epigenome-wide association studies are sensitive to such samples as they often present as outliers. Robust regression methods mitigate the impact of spurious outliers but are computationally intensive due to the high dimension of the data while least squares or maximum likelihood estimation remain popular choices. Finding and removing problematic samples during quality control is therefore an important first step of every epigenome-wide association study. We conducted a survey of publicly available DNA methylation datasets to see whether they suffer from the same quality issues that have been reported for gene expression datasets. Assuming that all samples included in datasets uploaded to the GEO repository were used in associated analyses, our results indicate that the current practice of quality control fails to detect many problematic samples that have the potential to severely bias findings.

Eleven percent of samples were flagged by at least one of the 17 control metrics defined by the microarray manufacturer. This does not mean that every flagged sample features inaccurate methylation levels and it is unclear how Illumina’s default thresholds were set and whether the resulting dichotomization is appropriate for flagging samples in all conditions. However, samples flagged by one or more criteria in control_metrics had substantially more outliers among the normally well-behaved SNP probes, an indication of low data quality, and therefore these samples require closer attention. In the specific case of monitoring bisulfite conversion, Zhou et al. suggested a more robust alternative to using the dedicated control probes [17].

As demonstrated on the example of monozygotic twins, the check_snp_agreement function does—at least in the absence of other issues—perfectly predict whether two samples come from the same person or not. The function is robust against SNP outliers, due to the soft classification scheme, whereas hard classification of genotypes as produced by k-means clustering or the use of fixed cutpoints in the *β*-value distribution can result in unexpected genotype mismatches. It is worth pointing out that the genetic fingerprint is the only way to detect mislabeling if a sample swap did not result in any apparent epitype/phenotype mismatch (e.g., two samples from the same sex). Mislabeling results in conflicts (unexpected disagreement between samples that are supposed to come from the same individual or unexpected agreement between samples that are supposed to come from different individuals), but it might be necessary to build upon further evidence in order to resolve conflicts and reassign the correct identities or even to narrow down which of the samples in conflict are the mislabeled ones. Furthermore, the utility of check_snp_agreement is limited for datasets that feature only a single sample per person.

In contrast, check_sex can be applied regardless of the number of samples available for each person. In our survey 1.6% of samples coming from 25% of the datasets were assigned the wrong sex. The actual rate of mislabeled samples is likely higher because sample swaps between donors of the same sex would have gone undetected. Assuming a balanced sex ratio and random mislabeling, only half of the potential mistakes would be captured by this test alone. If applicable, more comprehensive checks testing for correct tissue types and other features should be conducted. Regarding the few unclear cases in which the sex of the sample donors could not conclusively be inferred, we suspect that most of these samples suffer from other, possibly upstream issues and should be excluded. A few, however, might represent chromosomal disorders, e.g. Klinefelter syndrome, which has an reported incidence around 1 per 576 [18]. Because of their XXY genotype, individuals with Klinefelter syndrome, who are phenotypically male, would in Fig. 3 be located in the center of the upper right quadrant (for $\bar {T}^{X}_{n}$ on par with females, for $\bar {T}^{Y}_{n}$ on par with males).

Some samples showed evidence of contamination, especially those belonging to dataset **E**: we constructed two measures of sample contamination based on two subsets of probes, using either the average total intensities of probes targeting the Y chromosome $\left (\bar {T}^{Y}_{n}\right)$ or the *β*-values of the 65 SNP probes (*γ*_*n*_). The fact that both measures exhibited very high agreement, even though they were based on independent evidence and completely different principles, lends credence to our hypothesis of a single contamination source. In contrast, a strong correlation between *γ*_*n*_ and *O*_*n*_ (the average log odds of being an outlier across all 65 SNP probes) was to be expected, as both are derived from the same data. *O*_*n*_ is not a perfect proxy of contamination, and deviations from the trimodal distribution of *β*-values might also be caused by other issues, as evident when comparing samples passing and failing the control_metrics check. This does however not diminish the value of *O*_*n*_ as an overall sample quality indicator. This metric is easy to compute using our mixture modeling approach and can be applied regardless of the sex of the sample donors. Unlike a recently proposed metric of contamination of cord blood with maternal blood based on methylation probes [5], our measure snp_outliers is not dependent on the tissue type of the sample and contaminating DNA. Because the proportion of the 65 SNP probes expected to differ in a contaminated sample is a function of both the relative proportion of contamination and the number of SNPs for which the contaminating sample differs in genotype, it was not possible to determine a single cutoff for *O*_*n*_ above which samples should be classified as contaminated and excluded from further analysis. Judging by Fig. 4b however, we interpret that removing samples with an average log_2_ odds of − 4 represents a reasonable choice. Other authors have suggested to use the SNP probes as quality indicator before: Pidsley et al. proposed a metric quantifying the standard deviation of SNP probes in order to benchmark normalization methods for 450K datasets [13], but did not evaluate the quality of individual samples.

There are some limitations of our work. We did not check the associated publications for whether the authors of the original studies mentioned flagging or exclusion from downstream analyses of any samples from their datasets uploaded to the GEO repository. We advocate the inclusion of a simple tag in the metadata on GEO to indicate samples excluded in quality control steps, although no such indication was seen in the metadata of the studies we reviewed. Furthermore, our analysis is demonstrated exclusively on the Illumina Infinium HumanMethylation450 BeadChip, although the functions in our ewastools package work equally well on the newer Illumina Infinium MethylationEPIC BeadChip (commonly called 850K chip) for which far fewer datasets are currently available on GEO. While we excluded certain types of tissues in order to get a handle on the heterogeneity of datasets, this does not mean that these QC checks cannot be applied to those tissues, such as checking maternal contamination of fetal placenta. The selection of datasets was also restricted to those for which raw data were available, as this was needed to automate our QC testing, and this subset may not be representative of the entirety of > 1000 Illumina 450K methylation datasets on GEO. Nonetheless, we feel that our results indicate the need for additional QC checks as data quality issues appear prevalent in publicly available methylation datasets.

## Conclusion

Beyond the obvious measurement of methylation, a multitude of information can be inferred from high-dimensional DNA methylation data, information that can be checked for agreement with recorded covariates. This includes the use of principal component analysis to create lower-dimensional representations for discriminating between tissue types; comparing chronological and epigenetic age [19]; the estimation of cell proportions for blood samples, which can be as precise as actual blood cell counts [20]; or any other checks that might apply to the specific dataset at hand. We demonstrated in this work the high prevalence of failed and mislabeled samples in DNA methylation datasets and recommend that epigenome-wide association studies should start with comprehensive quality control. With ewastools, we provide a software package for the popular R programming language to conduct the quality checks described here. Existing R scripts do not have to be changed in order to incorporate these tests into existing analytic workflows. In addition, we recommend that researchers seeking to make their DNA methylation data available should also upload raw data in the form of.idat files. Taken together these steps will improve the reproducibility of epigenome-wide association studies.

## Abbreviations

450K: Illumina Infinium HumanMethylation450 BeadChip
CI: Confidence interval
EPIC: Illumina Infinium MethylationEPIC BeadChip
GEO: Gene expression omnibus
QC: Quality control
SNP: Single nucleotide polymorphism
